# Emotional distress among postpartum women in central Nepal: a cross-sectional study using structural equation modeling

**DOI:** 10.3389/fgwh.2026.1723556

**Published:** 2026-03-27

**Authors:** Miwako Okamoto, Sunila Shakya, Subasna Shrestha, Eiko Kaneda, Seira Kawada, Kenji Ohishi

**Affiliations:** 1 Faculty of Childhood Sport Education, Nippon Sport Science University, Tokyo, Japan; 2 Faculty of Obstetrics and Gynaecology, Dhulikhel Hospital, Kathmandu University School of Medical Sciences, Dhulikhel, Nepal; 3 Nursing and Midwifery Program, Kathmandu University School of Medical Sciences, Dhulikhel, Nepal; 4 Faculty of Sport Culture, Nippon Sport Science University, Tokyo, Japan; 5 Faculty of Sport Science, Nippon Sport Science University, Tokyo, Japan

**Keywords:** emotional distress, maternal mental health, Nepal, postpartum depression, risk factors, structural equation modelling

## Abstract

**Background:**

Postpartum emotional distress, including depression, anxiety, and stress, is a public health concern affecting maternal well-being and child development. Although several studies have identified risk factors among Nepalese women, little is known about the multifactorial pathways underlying postpartum emotional distress. This study aimed to determine the prevalence of postpartum emotional distress and to examine the pathways and interrelationships among factors associated with postpartum emotional distress among women in the Kathmandu Valley, Nepal.

**Methods:**

A hospital-based cross-sectional study was conducted at a tertiary hospital in central Nepal. A total of 381 postpartum women (4–12 weeks after delivery) were assessed using the Edinburgh Postnatal Depression Scale (EPDS) and the General Health Questionnaire-12 (GHQ-12). Sociodemographic, perinatal, childcare-related, and psychosocial data were collected. Exploratory factor analysis and structural equation modeling (SEM) were used to identify latent factors and the relationships among them.

**Results:**

The prevalence of postpartum emotional distress was 40.2% (95% CI: 35.2–45.3). Factor analysis identified seven latent factors: Childcare Experience, Mother's Economic Strength, Age of Parents, Extended Family Support, Emotional Distress, Help from Husband, and Satisfaction with Married Life. SEM demonstrated good fit (CFI = 0.971, RMSEA = 0.043). Satisfaction with Married Life (standardized coefficient: −0.66, *p* < 0.01), Help from Husband (−0.15, *p* < 0.01), Extended Family Support (−0.15, *p* < 0.05), and Mother's Economic Strength (−0.16, *p* < 0.05) were inversely associated with emotional distress.

**Conclusions:**

Postpartum emotional distress is highly prevalent among Nepalese women. Strengthening family and partner support, promoting community-based interventions, and enhancing routine mental health screening by health professionals are essential to both reduce distress and improve the psychological well-being of mothers and long-term outcomes for children.

## Introduction

Postpartum women are reported to be three times more likely to experience depression and other serious mental health problems than at any other time in their lives (1). Postpartum depression (PPD) and related conditions such as severe anxiety and loss of motivation not only compromise maternal health but also exert lasting effects on children's physical, cognitive, and emotional development (2, 57, 61). PPD has been linked to maternal suicide and child neglect, a form of child abuse (62, 63). More broadly, postpartum emotional distress, encompassing depression as well as other mental health problems, poses serious risks to mothers and infants. Preventing, identifying, and addressing such distress at an early stage are therefore critical to safeguarding maternal and child well-being. Evidence from studies of PPD further suggests that effective management can contribute to healthier brain development in children and positively influence personality formation, emotional regulation, and the development of social relationships (64).

The etiology of PPD is multifactorial, encompassing biological, psychological, and social determinants (65). Hormonal fluctuations during pregnancy, childbirth, and the postpartum period impose significant physiological stress (66), while the transition to motherhood brings psychological burdens related to new roles and responsibilities (67, 68). Social risk factors—including single motherhood, unintended pregnancy, marital conflict, lack of social support, and poverty—further compound vulnerability (62, 69, 70, 71). PPD is the most widely studied maternal mental health problem and can be considered as encompassed within the broader spectrum of emotional distress experienced during the postpartum period. Emotional distress encompasses depressive symptoms as well as anxiety, insomnia, stress, and persistent fatigue, which can impair maternal functioning, mother–child bonding, and child development (72–76). However, consensus is lacking on how best to define and conceptualize postpartum emotional distress (73, 76, 77). It is important to consider emotional distress more broadly to capture the diverse range of maternal mental health challenges, particularly in low- and middle-income countries where structural and social determinants strongly influence women's health.

Globally, the prevalence of PPD varies by region, being highest in the Middle East and lowest in Europe (3). In Nepal, estimates range from 17% to 33.7%, similar to those reported in neighboring South Asian countries (4–6, 78–80). Recent post-pandemic evidence from the region further suggests that postpartum depression remains highly prevalent; for example, a cross-sectional study in Bhutan reported substantial levels of PPD among mothers (7). Alarmingly, suicide was the leading cause of death among reproductive-age women in Nepal in 2009, and PPD may have been a contributing factor (66, 81). These findings underscore the need for enhanced maternal mental health support during pregnancy, childbirth, and postpartum care in Nepal.

Previous studies in Nepal have identified a wide range of risk factors for PPD, including single motherhood, unintended pregnancy, limited social support, poverty, inadequate rest during pregnancy, miscarriage, poor marital relationships, domestic violence, stressful life events, pregnancy complications, mode of delivery, infant gender, and infant health problems (4, 78, 80–82). Additional factors associated with postnatal anxiety include lack of continuous support from husbands, low maternal education, lower socioeconomic status, limited knowledge of infant care, and lack of childcare experience (6, 52).

Recent research has increasingly moved beyond identifying isolated risk factors for postpartum depression toward examining how psychosocial and contextual factors are interrelated. Pathway- and structural modeling approaches have been used to test hypothesized relationships among social support, stressors, and maternal depressive symptoms, demonstrating that multiple factors may operate through indirect pathways rather than independently. For example, Liu et al. (8) applied path model analysis to clarify relationships among key risk factors for postpartum depression among Chinese women. Such approaches offer a more integrated understanding of postpartum emotional distress and may help identify leverage points for prevention and early intervention.

Despite these findings, few studies have examined the multifaceted interrelationships among factors associated with postpartum emotional distress in Nepal. The present study therefore aimed to estimate the prevalence of postpartum emotional distress and to examine the pathways and associations among key psychosocial factors among women living in the Kathmandu Valley, providing insights relevant to maternal mental health policies in Nepal.

## Methods

### Study design and subjects

We conducted a hospital-based cross-sectional study at Dhulikhel Hospital (Kathmandu University Hospital), located in Dhulikhel Municipality, Kavre district, Nepal, between July 2019 and March 2020. The hospital is a tertiary care center serving approximately 1.9 million people from Kavrepalanchowk, Sindhupalchowk, Dolakha, Sindhuli, Ramechhap, Bhaktapur, and surrounding districts. The Department of Obstetrics and Gynecology provides services to pregnant women of diverse ethnic, socioeconomic, and educational backgrounds, thereby enhancing the representativeness of the study population. Eligible participants were postpartum women aged 18 years or older who were 4–12 weeks after delivery, had singleton live births (vaginal or caesarean), and had experienced no serious obstetrical complications. Women with multiple births, severe complications, or a documented history of mental illness were excluded. Postpartum women safely discharged with their newborns and attending the immunization and family planning units of the OBGYN department during their postnatal care visits were consecutively invited to participate. Written informed consent was obtained from each participant prior to the interview.

### Conceptual framework

**Figure 1:**
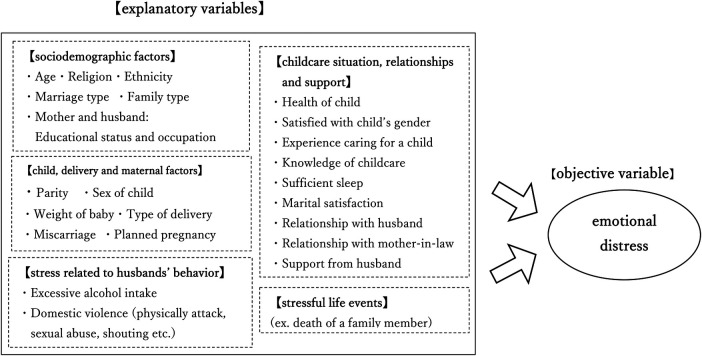
Conceptual framework of postpartum emotional distress. This figure illustrates the five domains and their hypothesized relationships with postpartum emotional distress.

We identified five domains of factors that may be associated with postpartum emotional distress through reviews of previous studies: sociodemographic characteristics, perinatal situations, childcare situations, stress related to husbands' behaviour, and stressful life events (65, 69, 70, 72) (4). Figure 1 presents the conceptual framework, which depicts the multifaceted interrelationships among these domains and guided the construction of a path diagram. This framework served as the basis for subsequent analyses using factor analysis and structural equation modelling (83).

### Sample size

We calculated the sample size to ensure a 95% confidence interval (CI) with a precision (d) of 5% for the estimated prevalence. Using the normal approximation formula, *n* *=* *Z²pq/d²*, with *Z* = 1.96, *p* denoting the expected prevalence, and *q* = 1−*p*, the required number was estimated. Assuming a 35% prevalence of psychological distress among postpartum mothers based on previous studies (4, 35), and allowing for a 10% non-response rate, the final sample size was determined to be 383. Finally, 381 mothers completed the questionnaires.

### Data collection procedure

Postpartum women safely discharged with their newborns and attending the immunization and family planning units of the OBGYN department during their postnatal care visits were consecutively invited to participate. Eligible participants received a verbal explanation of the study, and written informed consent was obtained prior to data collection. Data were collected through one-on-one interviews conducted by trained local research assistants in a private setting within the hospital to ensure confidentiality. Prior to the main survey, the questionnaire was pretested among 10 postpartum women at the same hospital who met the eligibility criteria. Trained local research assistants conducted interviews to assess clarity, comprehension, and cultural appropriateness of the items. Based on feedback from participants and discussion among the research team, minor wording adjustments were made, and the final version of the questionnaire was confirmed for use in the main study.

### Measurement tools

#### Postpartum emotional distress

Postpartum emotional distress was assessed using the Edinburgh Postnatal Depression Scale (EPDS) and the General Health Questionnaire-12 (GHQ-12) (9). The EPDS is a 10-item self-report instrument designed to screen for postpartum depression, with each item rated on a four-point Likert scale (0–3) covering a respondent's strongest feelings over the previous week (9), yielding a total score ranging from 0 to 30. Higher scores indicate greater depressive symptomatology. A systematic review has reported pooled sensitivity of 0.85 (95% CI: 0.79–0.90) and specificity of 0.84 (95% CI: 0.79–0.88) at a cut-off of ≥10 (38). The Nepali version of the EPDS has been validated and is recommended for postpartum depression screening in Nepal (4); therefore, we adopted the same cut-off in this study (4, 78). The GHQ-12 is a short version of the GHQ-60, developed to screen for common mental health problems, with six items addressing anxiety and depression and six items assessing social and occupational functioning (10, 29). Respondents rate each item on a four-point Likert scale (0 = not at all to 3 = much more than usual). For scoring, we applied the 0-0-1-1 method, resulting in a total score ranging from 0 to 12, with higher scores reflecting poorer mental health. The Nepali version of the GHQ-12 has been validated, and a cut-off score of ≥2 was used in this study (34, 40, 41). In the present study, internal consistency was acceptable for both instruments, with Cronbach's *α* of 0.777 for the EPDS and 0.735 for the GHQ-12.

#### Sociodemographic and perinatal characteristics

We collected information on participants' sociodemographic and perinatal characteristics, including maternal and spouse age, religion, ethnicity, type of marriage (arranged or not), family type (nuclear or extended), educational attainment (of both mother and spouse), occupation (housewife or paid employment), annual family income (> or ≤200,000 NPR), and respondent's personal income (> or ≤100,000 NPR). Perinatal variables included parity, child's sex and birth weight, mode of delivery, history of miscarriage, and whether the pregnancy was planned. For ethnicity, we applied a commonly used classification in Nepal, grouping participants as belonging to either advantaged or disadvantaged ethnic groups (4–6, 30, 43, 54).

#### Childcare and family relationships

We developed a 10-item questionnaire to assess participants' perceptions of childcare and family relationships. Each item was rated on a four-point Likert scale (1 = not at all to 4 = very much). The items covered the following aspects: the child's current health, satisfaction with the child's gender, prior experience in childcare, knowledge of child-rearing, adequacy of recent sleep, marital satisfaction (“Are you satisfied with your married life?”), relationship with husband (“Do you have a good relationship with your husband?”), relationship with mother-in-law (“Do you have a good relationship with your husband's mother?”), support from husband in child-rearing, and support from other family members in child-rearing.

#### Stress related to husbands' behavior and life events

We developed a questionnaire to assess stress related to husbands' behavior and stressful life events, consisting of six items rated on a four-point Likert scale (1 = not at all to 4 = very much). The items included: excessive alcohol intake (“Does your husband drink excessive amounts of alcohol?”); domestic violence (“Has your husband ever physically attacked you or thrown something at you that might hurt you?”); sexual assault (“Has your husband ever forced you to engage in sexual activity against your will?”); verbal or emotional abuse (“Has your husband ever insulted or shouted at you in a way that made you feel bad about yourself?”); working away from home (“Does your husband work away from home and leave the house for long periods of time?”); and other stressful life events (“Have you experienced stressful life events in recent years, such as the death of a family member?”).

#### Statistical analysis

Emotional distress was treated as a binary outcome for prevalence estimation and group comparisons, based on established cut-off scores of the EPDS and GHQ-12. For factor analysis and structural equation modelling, questionnaire items and derived latent variables were analyzed as continuous variables. We summarized participants' sociodemographic and perinatal characteristics, as well as scores on the EPDS and GHQ-12, using descriptive statistics. Emotional distress was defined as an EPDS score ≥10 or a GHQ-12 score ≥2. The prevalence of emotional distress and its 95% CI were estimated using the binomial distribution.

Comparisons between participants with emotional distress (ED group) and those without (non-ED group) were conducted using independent samples *t*-tests or Wilcoxon rank-sum tests for continuous variables, and Fisher's exact tests for categorical variables.

To identify underlying factors contributing to emotional distress, we conducted an exploratory factor analysis (EFA) on all 32 questionnaire items (96). Maximum-likelihood estimation with Promax rotation was applied, and items with factor loadings below 0.35 were removed. The EFA was then repeated to derive latent constructs for the hypothesized model. Based on these results, a hypothetical model was specified and evaluated using structural equation modelling (SEM). For model estimation, the maximum-likelihood method was applied, and standardized coefficients were reported. Model fit was assessed using the Goodness of Fit Index (GFI), Adjusted Goodness of Fit Index (AGFI), Comparative Fit Index (CFI), and Root Mean Square Error of Approximation (RMSEA). Akaike's Information Criterion (AIC) was used for comparative model evaluation (85, 96).

All statistical analyses were performed using IBM SPSS Statistics and Amos (version 29.0). A *p* value < 0.05 was considered statistically significant.

#### Ethical considerations

Ethical approval for this study was obtained from the Ethical Review Committee of the Kathmandu University School of Medical Sciences (Approval Number: 104/19). Written informed consent was obtained from all participants prior to enrolment. Data were collected through one-on-one interviews conducted by trained research assistants in a private setting within the hospital to ensure confidentiality.

## Results

### Participants' characteristics

Participants were predominantly young postpartum women, with a mean (SD) age of 26.4 (4.4) years, and the majority were Hindu, primiparous, and had completed higher education (Table 1). Socioeconomic characteristics varied, with most women being housewives and living in households with annual incomes above 200,000 NRS. Overall, 153 women met the criteria for emotional distress, yielding a prevalence of 40.2% (95% CI: 35.2–45.3%). Of these, 33 women scored above the cut-off on both the EPDS (≥10) and GHQ-12 (≥2), while 103 scored above the threshold on the EPDS alone and 17 on the GHQ-12 alone (Table 1).

**Table 1:** Characteristics of participants by emotional distress (ED) classification (*n* = 381).

Factors		Participants					
			G1: Non-ED		ED		
		*n* = 381	*n* = 228 (59.8%)		*n* = 153 (40.2%)		
				G2: EPDS ≥ 10 only	G3: GHQ-12 ≥ 2 only	G4: EPDS ≥ 10 & GHQ-12 ≥ 2	*p*
				*n* = 103 (27%)	*n* = 17 (4.5%)	*n* = 33 (8.7%)	
Age of mother (years)	(Mea*n* ± SD)	26.42 (4.35)	26.24 (4.23)	26.19 (4.36)	26.47 (3.79)	28.39 (5.03)	0.06^b^
Father (years)		29.33 (4.94)	29.03 (4.58)	29.19 (5.06)	30.76 (4.39)	31.18 (6.64)	0.07^b^
Religion	*n* (%)						0.18^a^
Hindu		297 (78.0)	183 (80.3)	73 (70.9)	11 (64.7)	30 (90.9)	
Non-Hindu		84 (22.0)	45 (19.7)	30 (29.1)	6 (35.3)	3 (9.1)	
Ethnicity	*n* (%)						0.08^a^
Advantaged ethnic group		134 (35.2)	88 (38.6)	28 (27.2)	4 (23.5)	14 (42.4)	
Disadvantaged ethnic group		247 (64.8)	140 (61.4)	75 (72.8)	13 (76.5)	19 (57.6)	
Parity	*n* (%)						0.07^a^
Primipara		220 (57.7)	140 (61.4)	61 (59.2)	10 (58.8)	9 (27.3)	
Multipara		161 (42.3)	88 (38.6)	42 (40.8)	7 (41.2)	24 (72.7)	
Educational status	*n* (%)						
Mother							<0.001^a^
Above secondary education		205 (53.8)	139 (61.0)	47 (45.6)	9 (52.9)	10 (30.3)	
Secondary education or below		176 (46.2)	89 (39.0)	56 (54.4)	8 (47.1)	23 (69.7)	
Father							0.02^a^
Above secondary education		215 (56.6)	139 (61.0)	55 (53.9)	9 (52.9)	12 (36.4)	
Secondary education or below		165 (43.4)	89 (39.0)	47 (46.1)	8 (47.1)	21 (63.6)	
Mother's occupation	*n* (%)						0.52^a^
Housewife		303 (79.5)	177 (77.6)	84 (81.6)	13 (76.5)	29 (87.9)	
Non-housewife		78 (20.5)	51 (22.4)	19 (18.4)	4 (23.5)	4 (12.1)	
Mother's annual income (NRR)	*n* (%)						0.09^a^
>100,000		70 (18.4)	48 (21.1)	17 (16.5)	2 (11.8)	3 (9.1)	
≤100,000		311 (81.6)	180 (78.9)	86 (83.5)	15 (88.2)	30 (90.9)	
Family annual income (NRR)	*n* (%)						0.008^a^
>200,000		307 (80.6)	191 (83.8)	78 (75.7)	14 (82.4)	24 (72.7)	
≤200,000		74 (19.4)	37 (16.2)	25 (24.3)	3 (17.6)	9 (27.3)	
Marriage arrangement	*n* (%)						0.51^a^
By family		233 (61.2)	145 (63.6)	61 (59.2)	8 (47.1)	19 (57.6)	
Self-arranged		148 (38.8)	83 (36.4)	42 (40.8)	9 (52.9)	14 (42.4)	
Family type	*n* (%)						0.31^a^
Nuclear		148 (38.8)	82 (36.0)	41 (39.8)	9 (52.9)	16 (48.5)	
Joint		233 (61.2)	146 (64.0)	62 (60.2)	8 (47.1)	17 (51.5)	
Planned pregnancy	*n* (%)						0.006^a^
Yes		346 (90.8)	215 (94.3)	85 (82.5)	15 (88.2)	31 (93.9)	
No		35 (9.2)	13 (5.7)	18 (17.5)	2 (11.8)	2 (6.1)	
History of miscarriage	*n* (%)						0.09^a^
None		344 (89.8)	209 (91.7)	93 (90.3)	14 (82.4)	26 (78.8)	
One or more		39 (10.2)	19 (8.3)	10 (9.7)	3 (17.6)	7 (21.2)	
Complications during pregnancy	*n* (%)						0.59^a^
No		252 (66.1)	146 (64.0)	69 (67.0)	13 (76.5)	24 (72.7)	
Yes		129 (33.9)	82 (36.0)	34 (33.0)	4 (23.5)	9 (27.3)	
Mode of delivery	*n* (%)						0.69^a^
Vaginal		237 (62.2)	142 (62.3)	63 (61.2)	9 (52.9)	23 (69.7)	
Caesarean section		144 (37.8)	86 (37.7)	40 (38.8)	8 (47.1)	10 (30.3)	
Sex of last child	*n* (%)						0.14^a^
Female		167 (43.8)	100 (43.9)	46 (44.7)	11 (64.7)	10 (30.3)	
Male		214 (56.2)	128 (56.1)	57 (55.3)	6 (35.3)	23 (69.7)	
Weight of the child (g) (Mean ± SD)		2,958.33 (459.95)	2,969.17 (443.07)	2,917.51 (467.91)	2,917.94 (400.39)	3,031.66 (473.58)	0.59^b^

^a^ Fisher's exact test

^b^ One-way analysis of variance. G1 = Non-ED; G2 = EPDS ≥ 10 only; G3 = GHQ-12 ≥ 2 only; G4 = EPDS ≧ 10 & GHQ-12 ≥ 2. NRR = Nepalese Rupees.

As shown in Table 1, emotional distress was significantly associated with lower maternal education (*p* < 0.001), lower spousal education (*p* = 0.02), lower family income (*p* = 0.008), and unplanned pregnancy (*p* = 0.006).

### Supplementary analyses of group differences

Supplementary analyses indicated that women with emotional distress differed from those without emotional distress in several aspects of childcare situations, family relationships, and stress related to husbands' behavior. Specifically, women with emotional distress were less likely to report sufficient sleep, support from their husbands or other family members, and satisfaction with married life, and were more likely to report problematic alcohol use and psychological abuse by their husbands. Detailed results of these analyses are presented in Supplementary Table S1.

### Factor structure of the variables investigated

An initial exploratory factor analysis (EFA) of 34 questionnaire items, including summary scores of the EPDS and GHQ-12, suggested seven underlying constructs. After excluding 17 items with factor loadings <0.35, a subsequent EFA with Promax rotation confirmed a seven-factor structure, accounting for 61.9% of the cumulative variance. Table 2 presents the extracted latent factors, their corresponding items, and factor loadings. The extracted factors were labeled as follows: Child-caring Experience, Mother's Economic Strength, Age of Parents, Extended Family Support, Emotional Distress, Receive Help from Husband, and Satisfaction with Married Life.

**Table 2:** Factor loadings and inter-factor correlations from exploratory factor analysis.

Latent variable	I	II	III	IV	V	VI	VII
Child-caring Experience							
Before you gave birth, did you have experience caring for a baby? 【X1】	0.99	0.01	−0.04	0.03	−0.03	0.01	−0.01
Do you have knowledge of childcare? 【X2】	0.91	0.00	−0.07	0.01	−0.01	−0.01	−0.01
Parity 【X3】	0.63	−0.02	0.19	−0.08	0.04	0.02	0.03
Mother's Economic Strength							
Mother's income 【X4】	0.01	0.99	−0.02	−0.01	−0.01	−0.01	−0.02
Occupation of mother 【X5】	−0.01	0.89	0.02	0.03	−0.01	0.01	0.02
Age of Parents							
Age of mother 【X6】	−0.00	0.04	0.93	0.02	−0.02	0.02	0.03
Age of father 【X7】	0.00	−0.04	0.76	−0.02	0.01	−0.02	−0.05
Extended Family Support							
Do you receive help from other family members (besides your husband) in raising children? 【X8】	−0.01	−0.04	0.04	0.98	−0.03	0.02	0.02
Family type 【X9】	0.01	0.06	−0.04	0.68	−0.02	−0.01	−0.04
Emotional Distress							
GHQ 【GHQ】	−0.00	0.04	−0.01	0.02	0.99	−0.02	−0.00
EPDS 【EPDS】	−0.02	−0.06	0.00	−0.08	0.50	0.06	−0.03
Receive Help from Husband							
Does your husband work away from home and leave the house for long periods of time? 【X10】	−0.01	0.03	−0.01	−0.06	−0.06	0.99	−0.05
Do you receive help from your husband in raising children? 【X11】	−0.04	0.05	−0.01	−0.09	−0.12	−0.50	−0.08
Satisfaction with Married Life							
Has your husband ever insulted or shouted at you in a way that made you feel bad about yourself?【X12】	−0.05	0.02	−0.02	−0.05	−0.01	−0.03	0.57
Are you satisfied with the married life you have with your husband? 【X13】	−0.04	0.05	0.05	−0.04	−0.02	−0.07	−0.45
Does your husband drink excessive amounts of alcohol?【X14】	−0.04	0.00	0.02	−0.05	0.01	−0.11	0.43
Do you have a good relationship with your husband?【X15】	0.03	−0.05	−0.02	−0.03	−0.11	−0.14	−0.39
Factor correlation matrix	I	II	III	IV	V	VI	VII
I	—	−0.02	0.42	−0.30	0.07	0.02	0.26
II		—	0.16	0.12	−0.14	0.02	0.15
III			—	−0.22	0.15	−0.03	0.27
IV				—	−0.17	0.08	−0.09
V					—	0.10	0.26
VI						—	0.02
VII							—
Sum of squares of loads accumulation (%)	8.75	25.64	33.31	44.48	51.30	57.63	61.89

【 】: name of observed variable.

Extraction method: Maximum likelihood; Rotation method: Promax, only factor loadings ≥0.35 are shown.

### Structural equation modeling

**Figure 2:**
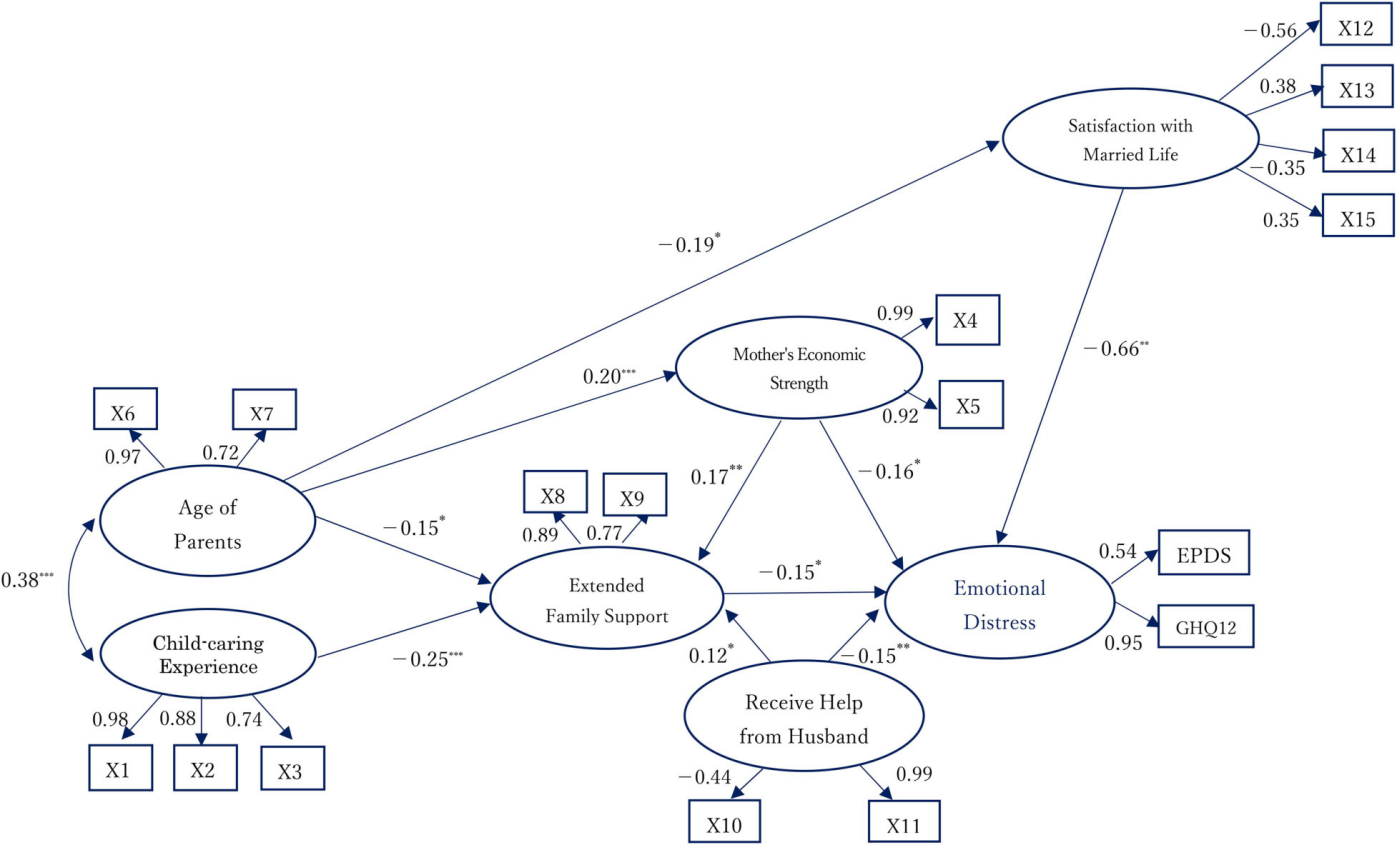
Structural equation model of postpartum emotional distress. This model illustrates the inter-relationships among seven latent factors. Standardized path coefficients are shown, with solid arrows indicating statistically significant paths (*p* < 0.05). Significance levels: *p < 0.05; **p < 0.01; ***p < 0.001. Model fit indices: GFI = 0.948, AGFI = 0.925, CFI = 0.971, RMSEA = 0.043, AIC = 273.643.

Based on the hypothesized conceptual framework, a structural equation model was constructed to examine the inter-relationships among the seven latent factors identified in the exploratory factor analysis (Figure 2). The model demonstrated good fit with the data (GFI = 0.948, AGFI = 0.925, CFI = 0.971, RMSEA = 0.043) and yielded the lowest Akaike's Information Criterion (AIC = 273.643) compared with alternative models.

In the final model, Mother's Economic Strength (standardized coefficient: −0.16, *p* < 0.05), Extended Family Support (–0.15, *p* < 0.05), Receive Help from Husband (–0.15, *p* < 0.01), and Satisfaction with Married Life (–0.66, *p* < 0.01) were negatively associated with Emotional Distress. Child-caring Experience and Age of Parents showed a significant covariance (standardized coefficient: 0.38, *p* < 0.001) and were indirectly associated with Emotional Distress. Child-caring Experience was related to Extended Family Support (–0.25, *p* < 0.001), which in turn reduced Emotional Distress (–0.15, *p* < 0.05). Age of Parents influenced Emotional Distress indirectly through Extended Family Support (–0.15, *p* < 0.05), Mother's Economic Strength (0.20, *p* < 0.001), and Satisfaction with Married Life (–0.19, *p* < 0.05). Extended Family Support also acted as a mediator between Mother's Economic Strength and Support from Husband in their effects on Emotional Distress.

Standardized direct, indirect, and total effects from the SEM are summarized in Table 3. Several latent variables showed significant direct and/or indirect associations with emotional distress. In particular, satisfaction with married life exhibited the strongest total effect on emotional distress, while support from husbands and extended family support influenced emotional distress primarily through indirect pathways.

**Table 3:** Summary of the direct, indirect and total effects on emotional distress.

Latent variable	Effects		
	Direct	Indirect	Total
Child-caring Experience	0.000	−0.031	−0.031
Mother's Economic Strength	−0.157	−0.021	−0.178
Age of Parents	0.000	−0.104	−0.104
Extended Family Supporters	−0.146	0.000	−0.146
Receive Help from Husband	−0.148	0.014	−0.134
Satisfaction with Married Life	−0.658	0.000	−0.658

Detailed results of mean difference tests are provided in Supplementary Table S1. Correlation matrices are presented in Supplementary Supplementary Table S2.

## Discussion

This study identified a high prevalence of postpartum emotional distress among women in central Nepal and showed that modifiable and non-modifiable factors were interrelated through complex pathways, as examined using structural equation modeling. Marital satisfaction exhibited the strongest direct association with emotional distress, while support from husbands and extended family was primarily linked through indirect pathways. In addition, non-modifiable factors such as parental age, childcare experience, and maternal economic strength affected emotional distress indirectly via social and relational mechanisms. These findings help to contextualize how relational, social, and structural factors are associated with postpartum emotional distress in this setting.

### Interpretation of pathways and interrelationships

The present findings have important implications for clinical practice and maternal health policy in low- and middle-income countries such as Nepal. Using structural equation modeling, this study helped to clarify how multiple factors associated with postpartum emotional distress are interconnected. The strong direct association between marital satisfaction and emotional distress suggests that the quality of the marital relationship is a key factor associated with maternal mental health in the postpartum period. In contrast, support from husbands and extended family primarily operated through indirect pathways, indicating that these forms of support may buffer emotional distress by improving relational stability and daily caregiving conditions rather than exerting immediate effects.

These findings are consistent with pathway-based studies conducted in other Asian settings. For example, Liu et al. (8) reported that marital relationships and social support influenced postpartum depression both directly and indirectly through interconnected pathways among Chinese women. Similar to our findings, their model highlighted the importance of relational factors over isolated demographic variables. However, the relative strength of marital satisfaction observed in the present study may reflect the sociocultural context of Nepal, where marital and extended family relationships play a particularly central role in women's daily lives during the postpartum period. Our findings align with recent evidence from South Asia. Kapoor et al. (11), in a tertiary care setting in India, also reported that interpersonal and family-related factors were strongly associated with postpartum depression. Together, these findings suggest that postpartum care may benefit from moving beyond symptom screening alone by paying greater attention to marital relationships, partner involvement, and family support structures.

Incorporating family-centered approaches into perinatal mental health services—such as partner-inclusive education, screening for domestic violence, and linkage to community support networks—may contribute reduce postpartum emotional distress. Structural equation modeling provides a useful framework for identifying such intervention points and for designing integrated, context-sensitive prevention strategies.

### Prevalence of postpartum emotional distress

The prevalence of psychological burden among postpartum women varies depending on its definition and measurement. Two recent studies in Nepal used the EPDS with the same cut-off value of ≥10, reporting prevalence rates of 30% (95% CI: 26%–35%) and 34% (95% CI: 29%–39%), respectively (4, 78). In the present study, the prevalence was slightly higher at 40% (95% CI: 35%–45%), which may be explained by the inclusion of the GHQ-12 in addition to the EPDS for assessing emotional distress. Notably, 17 of 153 women identified as having emotional distress were captured by the GHQ-12 only. This suggests that combining the two instruments may facilitate the detection of not only depression but also a broader range of maternal mental health problems during the perinatal period. It should be noted that differences in cut-off values and assessment strategies across studies complicate direct comparisons of prevalence estimates. The EPDS has been used with varying cut-off scores ranging from ≥9 to ≥13 in different settings, and the GHQ-12 is likewise subject to heterogeneity in scoring methods and thresholds. Moreover, studies relying on a single screening instrument may capture depressive symptoms differently from those employing multiple instruments to assess broader emotional distress. Therefore, variations in prevalence rates across studies should be interpreted with caution, as they may reflect methodological differences rather than true differences in underlying psychological burden.

### Modifiable risk factors

Learning about the characteristics associated with emotional distress is an important step for health professionals in two ways. First, such knowledge facilitates the early identification of women at high risk. Relevant characteristics include low educational attainment, poor economic status (low household income), limited family support, stress related to partner behavior, unplanned pregnancy, reduced sleep quality after delivery, and experiences of domestic violence (5, 6, 25, 33, 53, 54).

In particular, Nepalese women are at risk of reduced sleep quality because they are likely to return to work shortly after delivery and also responsible alone for childcare and housework without any help from supporters.

Second, recognizing modifiable risk characteristics can lead to effective measures to alleviate the burden. For example, enhancing support from family members and encouraging a partner in his behavioral changes would be actionable management. Nonetheless, non-modifiable characteristics can also be vital because they may be associated with modifiable ones. In this regard, it is helpful for health professionals to appreciate the inter-relationships among the factors associated with emotional distress.

Of the six factors directly or indirectly associated with emotional distress identified in the present study, three were modifiable: *Extended Family Support*, *Receive Help from Husband*, and *Satisfaction with Married Life*. It is vital not to leave a woman alone, particularly in child-rearing and housework, during pregnancy and after delivery. Therefore, health professionals engaging in perinatal care need to repeatedly assess a woman's circumstances to see whether she is getting any support from her family including husband or neighbors. They also should prepare to manage undesirable situations, if any, by collaborating with relevant people. Coparenting, where parents cooperate with other family members, is a key to coping with difficulties encountered in child-rearing. Of note, grandparent support could lead young, anxious parents to psychological well-being in Asian countries where interdependence culture is pervasive (51).

*Satisfaction with Married Life* exerted the largest impact on emotional distress with path coefficient of −0.66. It can also be modifiable because a husband's habits, reflected in items X12 (verbal or emotional abuse) and X14 (alcohol drinking), are critical determinants of the construct. Partner violence against women poses significant physical and mental health problems in perinatal care (Campbell, 2002). A systematic review estimated the overall prevalence of continuous violence against postpartum women at 29.3% (95% CI: 23.3–35.3%) (37). The review also revealed that the prevalence depends on country income: 35.1% for low-middle income, 17.4% for low-income, and 3.3% for high-income countries (37). In fact, Bhatta et al. reported that the prevalence of domestic violence in Nepal against women during pregnancy and the postpartum period was 26.2%, and it was much higher in the second (34.6%) and the third (32.7%) trimester of pregnancy (19). Characteristics associated with domestic violence included 2–5 years of marriage life compared with <2 years, low husbands' education status, high mother-in-law's controlling behavior, and previous history of domestic violence (19). Furthermore, pregnant women residing in urban areas, older age, uneducated, and poor socioeconomic status were more likely to be victims of assault (31). A prospective cohort study in Nepal by Budhathoki et al. showed that postpartum depression was observed in 100% of women with poor communication or conversation with their husbands, while 17% of those with good conversations with partners (23).

These findings highlight the urgent need for preventive strategies in perinatal health systems, particularly through parent education classes that involve not only primiparous but also multiparous women and their family members. Such programs should include guidance on childcare, life after childbirth, prevention of domestic violence, and routine mental health screening. Health professionals must be prepared to detect early signs of marital conflict by listening carefully to pregnant women and using validated instruments for mental health and domestic violence. Furthermore, they should be equipped to provide referral information for counseling or safe shelter to ensure the safety of mothers and infants. By continuously assessing women's circumstances and promoting supportive involvement of partners and family members, health systems may help reduce the risk of postpartum emotional distress and improve maternal psychological well-being.

### Non-modifiable factors

The remaining three factors—*Childcare Experience, Parental Age,* and *Mother's Economic Strength*—were non-modifiable but exerted direct or indirect influences on emotional distress. Greater childcare experience and older parental age were associated with lower levels of social support, which in turn negatively affected mental health. This is consistent with evidence that multiparous women report less social support and marital satisfaction compared with primiparous women (56). Such women may receive less help from relatives or neighbors, who may assume that experienced mothers require little support. Although childbirth experience itself cannot be modified, mothers' childcare knowledge can be improved through education programs delivered by health professionals.

*Mother's Economic Strength* emerged as a protective factor, as women with greater financial resources were less likely to experience emotional distress, both directly and indirectly through enhanced social support. Conversely, women with limited economic means were more vulnerable to psychological burden. These findings are consistent with recent evidence indicating that socioeconomic disadvantage and restricted access to supportive resources remain key determinants of perinatal mental health in low- and middle-income countries (12, 13).

From a policy and clinical perspective, these results suggest the potential value of integrating perinatal mental health support with broader social protection and maternal education initiatives, particularly in resource-constrained settings. Enhancing community-based support systems and improving access to educational resources for mothers may help to lessen the impact of non-modifiable structural vulnerabilities on postpartum emotional distress (14).

### Limitation and strength of the study

The main strength of this study lies in its use of factor analysis and structural equation modeling, which enabled us to examine inter-relationships among latent variables that would not have been captured by conventional multivariable approaches, where each variable is treated only as a predictor or an outcome. However, several limitations should be noted. First, due to the cross-sectional design, causal directions cannot be firmly established. For instance, a woman's depressive mood might also influence her satisfaction with married life or the extent of support she receives. Nonetheless, interpreting associations within the framework of accumulated knowledge is a core principle of structural equation modeling (83). Second, we did not collect data on depression during pregnancy or coexisting mental health problems, which might have provided additional insights. Third, social desirability bias and recall bias may have influenced participants' responses to the survey items. Fourth, the findings may not be fully generalizable beyond the study setting, as participants were recruited from a single hospital in the Kathmandu Valley of Nepal. Finally, our data were collected before the COVID-19 pandemic; changes in social support systems and stressors during the pandemic may affect the applicability of these findings to current conditions.

## Conclusions

The prevalence of postpartum emotional distress among Nepalese women was about 40% in this study. Factor analysis and structural equation modeling identified both modifiable and non-modifiable factors and clarified their inter-relationships. Modifiable factors—family support, help from husbands, and marital satisfaction—emerged as key intervention targets, while non-modifiable factors such as parental age, childcare experience, and economic strength influenced maternal mental health indirectly through social support. These findings underscore the need for perinatal mental health programs in Nepal that extend beyond treating depressive symptoms to strengthening family and community support, fostering partner involvement, and promoting women's socioeconomic empowerment. A holistic approach addressing both modifiable and non-modifiable factors is essential to safeguard the mental health of mothers and children.

## Data Availability

The original contributions presented in the study are included in the article/Supplementary Material, further inquiries can be directed to the corresponding author.

## References

[1] World Health Organization. Maternal Mental Health and Child Health and Development in Low and Middle Income Countries: Report of the Meeting. Geneva: WHO (2008).

[2] Closa-Monasterolo R Gispert-Llaurado M Canals J Luque V Zaragoza-Jordana M Koletzko B The effect of postpartum depression and current mental health problems of the mother on child behaviour at eight years. Matern Child Health J. (2017) 21(7):1563–72. 10.1007/s10995-016-2255-4 28188472

[3] Shorey S Chee CYI Ng ED Chan YH Tam WWS Chong YS. Prevalence and incidence of postpartum depression among healthy mothers: a systematic review and meta-analysis. J Psychiatr Res. (2018) 104:235–48. 10.1016/j.jpsychires.2018.08.001 30114665

[4] Giri RK Khatri RB Mishra SR Khanal V Sharma VD Gartoula RP. Prevalence and factors associated with depressive symptoms among postpartum mothers in Nepal. BMC Res Notes. (2015) 8:111. 10.1186/s13104-015-1074-3 25885925 PMC4383200

[5] Ho-Yen SD Bondevik GT Eberhard-Gran M Bjorvatn B. Factors associated with depressive symptoms among postnatal women in Nepal. Acta Obstet Gynecol Scand. (2007) 86(3):291–7. 10.1080/00016340601110812 17364302

[6] Shrestha S Adachi K Petrini MA Shrestha S. Factors associated with postnatal anxiety among primiparous mothers in Nepal. Int Nurs Rev. (2014) 61(3):427–34. 10.1111/inr.12109 25039801

[7] Zangmo S Boonchieng W Suvanayos C Gyeltshen K Siewchaisakul P. Prevalence and factors associated with postpartum depression among bhutanese mothers: a cross-sectional study. Womens Health Nurs. (2024) 30:238–49. 10.4069/whn.2024.09.02 39385550 PMC11467251

[8] Liu S Yan Y Gao X Xiang S Sha T Zeng G Risk factors for postpartum depression among Chinese women: path model analysis. BMC Pregnancy Childbirth. (2017) 17:133. 10.1186/s12884-017-1320-2 28464884 PMC5414210

[9] Cox JL Holden JM Sagovsky R. Detection of postnatal depression. Development of the 10-item Edinburgh postnatal depression scale. Br J Psychiatry. (1987) 150:782–6. 10.1192/bjp.150.6.782 3651732

[10] Goldberg DP Gater R Sartorius N Ustun TB Piccinelli M Gureje O The validity of two versions of the GHQ in the WHO study of mental illness in general health care. Psychol Med. (1997) 27(1):191–7. 10.1017/S0033291796004242 9122299

[11] Kapoor B Malik N Gupta G Khan IA. A cross-sectional study exploring postpartum depression at a tertiary care center in Eastern Uttar Pradesh, India. Cureus. (2024) 16(4):e58653. 10.7759/cureus.58653 38770470 PMC11104705

[12] Fisher J de Mello MC Patel V Rahman A Tran T Holton S Prevalence and determinants of common perinatal mental disorders in women in low- and lower-middle-income countries: a systematic review. Bull W H O. (2012) 90:139–49. 10.2471/BLT.11.091850 PMC3302553 22423165

[13] McNab SE Dryer SL Fitzgerald L Gomez P Bhatti AM Kenyi E The silent burden: a landscape analysis of common perinatal mental disorders in low- and middle-income countries. BMC Pregnancy Childbirth. (2022) 22:458. 10.1186/s12884-022-04589-z 35443652 PMC9019797

[14] World Health Organization. World Mental Health Report: Transforming Mental Health for All. Geneva: World Health Organization (2022). Available online at: https://www.who.int/publications/i/item/9789240049338 (Accessed August 01, 2025).

[15] Alkan Ö Sercemeli C Özmen K. Verbal and psychological violence against women in Turkey and its determinants. PLoS One. (2022) 17(10):e0275950. 10.1371/journal.pone.0275950 36215284 PMC9550074

[16] Alshikh Ahmad H Alkhatib A Luo J. Prevalence and risk factors of postpartum depression in the Middle East: a systematic review and meta-analysis. BMC Pregnancy Childbirth. (2021) 21(1):542. 10.1186/s12884-021-04016-9 34362325 PMC8343347

[17] Assal-Zrike S Marks K Atzaba-Poria N. Maternal postpartum emotional distress and preterm social withdrawal in the Bedouin culture. Res. Child Adolesc. Psychopathol. (2022) 50(7):907–18. 10.1007/s10802-022-00928-y 35098419

[18] Barnes J Theule J. Maternal depression and infant attachment security: a meta-analysis. Infant Ment Health J. (2019) 40(6):817–34. 10.1002/imhj.21808 31415711

[19] Bhatta N Assanangkornchai S. Patterns of domestic violence against women during pregnancy and the postpartum period in Kathmandu, Nepal. Asia Pac Psychiatry. (2019) 11(1):e12342. 10.1111/appy.12342 30474319

[20] Bhusal BR Bhandari N. Identifying the factors associated with depressive symptoms among postpartum mothers in Kathmandu, Nepal. Int J Nurs Sci. (2018) 5(3):268–74. 10.1016/j.ijnss.2018.06.001 31406836 PMC6626200

[21] Bhusal BR Bhandari N Chapagai M Gavidia T. Validating the Edinburgh postnatal depression scale as a screening tool for postpartum depression in Kathmandu, Nepal. Int J Ment Health Syst. (2016) 10:71. 10.1186/s13033-016-0102-6 27785152 PMC5073833

[22] Brandon DH Tully KP Silva SG Malcolm WF Murtha AP Turner BS Emotional responses of mothers of late-preterm and term infants. J Obstet Gynecol Neonatal Nurs. (2011) 40(6):719–31. 10.1111/j.1552-6909.2011.01290.x 22092914 PMC4074409

[23] Budhathoki N Dahal M Bhusal S Ojha H Pandey S Basnet S. Violence against women by their husband and postpartum depression. J Nepal Health Res Counc. (2012) 10(22):176–80. 23281446

[24] Ceriani Cernadas JM. Postpartum depression: risks and early detection. Arch Argent Pediatr. (2020) 118(3):154–5. 10.5546/aap.2020.eng.154 32470247

[25] Chalise A Bhandari TR. Postpartum depression and its associated factors: a community-based study in Nepal. J Nepal Health Res Counc. (2019) 17(2):200–5. 10.33314/jnhrc.v17i2.1443 31455934

[26] Dawadi P Bhatta AS Shakya J. Factors associated with postpartum depressive symptoms in community of central Nepal. Psychiatry J. (2020) 2020:8305304. 10.1155/2020/8305304 32318592 PMC7165355

[27] Department of Health Services, Government of Nepal. Nepal Maternal Mortality and Morbidity Study (MMMS) 2008/2009. Kathmandu: Family Health Division, Department of Health Services (2010). Available online at: https://km.mohp.gov.np/sites/default/files/201807/NMMM_Study_2008_09.pdf (Accessed August 01, 2025).

[28] Earls MF. Incorporating recognition and management of perinatal and postpartum depression into pediatric practice. Pediatrics. (2010) 126(5):1032–9. 10.1542/peds.2010-2348 20974776

[29] Goldberg DP Blackwell B. Psychiatric illness in general practice: a detailed study using a new method of case identification. Br Med J. (1970) 1(5707):439–43. 10.1136/bmj.2.5707.439 5420206 PMC1700485

[30] Government of Nepal, National Planning Commission Secretariat. Population Monograph of Nepal. Volume II: Social Demography. Kathmandu: Central Bureau of Statistics (2014).

[31] Habib S Abbasi N Khan B Danish N Nazir Q. Domestic violence among pregnant women. J Ayub Med Coll Abbottabad. (2018) 30(2):237–40. 29938426

[32] Ingadóttir E Thome M. Evaluation of a web-based course for community nurses on postpartum emotional distress. Scand J Caring Sci. (2006) 20(1):86–92. 10.1111/j.1471-6712.2006.00383.x 16489964

[33] Khadka R Hong SA Chang YS. Prevalence and determinants of poor sleep quality and depression among postpartum women: a community-based study in Ramechhap district, Nepal. Int Health. (2020) 12(2):125–31. 10.1093/inthealth/ihz071 31294785 PMC7057136

[34] Koirala NR Regmi SK Sharma VD Khalid A. Sensitivity and validity of the general health questionnaire (GHQ-12) in a rural community setting in Nepal. Nepal J Psychiatry. (1999) 1(1):34–40.

[35] Koirala P Chuemchit M. Depression and domestic violence experiences among Asian women: a systematic review. Int J Women’s Health. (2020) 12:21–35. 10.2147/IJWH.S235864 32021490 PMC6970613

[36] Kumwar D Corey EK Sharma P Risal A. Screening for postpartum depression and associated factors among women who deliver at a university hospital, Nepal. Kathmandu Univ Med J. (2015) 13(49):44–8. 10.3126/kumj.v13i1.13752 26620748

[37] Lamaro T Enqueselassie F Deyessa N Burusie A Dessalegn B Sisay D. The pooled prevalence of perinatal partner violence against postpartum women for index child: a systematic review and meta-analysis. Heliyon. (2023) 9(4):e15119. 10.1016/j.heliyon.2023.e15119 37089356 PMC10113858

[38] Levis B Negeri Z Sun Y Benedetti A Thombs BD, DEPRESsion Screening Data (DEPRESSD) EPDS Group. Accuracy of the Edinburgh postnatal depression scale (EPDS) for screening to detect major depression among pregnant and postpartum women: systematic review and meta-analysis of individual participant data. Br Med J. (2020) 371:m4022. 10.1136/bmj.m4022 33177069 PMC7656313

[39] Marahatta K Samuel R Sharma P Dixit L Shrestha BR. Suicide burden and prevention in Nepal: the need for a national strategy. WHO South East Asia J Public Health. (2017) 6(1):45–9. 10.4103/2224-3151.206227 28597859

[40] Maridal HK Bjørgaas HM Hagen K Jonsbu E Mahat P Malakar S Psychological distress among caregivers of children with neurodevelopmental disorders in Nepal. Int J Environ Res Public Health. (2021) 18(5):2460. 10.3390/ijerph18052460 33801567 PMC7967590

[41] McDowell I. Measuring Health: A Guide to Rating Scales and Questionnaires. 3rd ed. New York: Oxford University Press (2006).

[42] Mercer RT. Becoming a Mother. New York: Springer Publishing (1995).

[43] National Indigenous Women’s Federation. Economic Empowerment of Indigenous Women in Nepal. Kathmandu: NIWF (2018).

[44] Navarro P Ascaso C Garcia-Esteve L Aguado J Torres A Martín-Santos R. Postnatal psychiatric morbidity: a validation study of the GHQ-12 and the EPDS as screening tools. Gen Hosp Psychiatry. (2007) 29(1):1–7. 10.1016/j.genhosppsych.2006.10.004 17189737

[45] Norman GR Streiner DL. PDQ Statistics. 3rd ed. Hamilton: BC Decker (2003).

[46] Norman GR Streiner DL. Biostatistics: The Bare Essentials. 4th ed. North Carolina: People’s Medical Publishing House–USA (2014).

[47] Oztora S Arslan A Caylan A Dagdeviren HN. Postpartum depression and affecting factors in primary care. Niger J Clin Pract. (2019) 22(1):85–91. 10.4103/njcp.njcp_118_18 30666025

[48] Page LA. The New Midwifery: Science and Sensitivity in Practice. London: Churchill Livingstone (2002).

[49] Pearl J. The causal foundations of structural equation modelling. In: Hoyle RH, editor. Handbook of Structural Equation Modeling. New York: Guilford Press (2012). p. 68–91.

[50] Pearlstein T Howard M Salisbury A Zlotnick C. Postpartum depression. Am J Obstet Gynecol. (2009) 200(4):357–64. 10.1016/j.ajog.2008.11.033 19318144 PMC3918890

[51] Poblete AT Gee CB. Partner support and grandparent support as predictors of change in coparenting quality. J Child Fam Stud. (2018) 27(7):2295–304. 10.1007/s10826-018-1069-5 30505139 PMC6258038

[52] Sapkota S Kobayashi T Takase M. Impact on perceived postnatal support, maternal anxiety and symptoms of depression in new mothers in Nepal when their husbands provide continuous support during labour. Midwifery. (2013) 29(11):1264–71. 10.1016/j.midw.2012.11.010 23415367

[53] Shi P Ren H Li H Dai Q. Maternal depression and suicide at immediate prenatal and early postpartum periods and psychosocial risk factors. Psychiatry Res. (2018) 261:298–306. 10.1016/j.psychres.2018.01.002 29331710

[54] Singh DR Sunuwar DR Adhikari S Singh S. Determining factors for the prevalence of depressive symptoms among postpartum mothers in lowland region in southern Nepal. PLoS One. (2021) 16(1):e0245199. 10.1371/journal.pone.0245199 33481863 PMC7822291

[55] Slomian J Honvo G Emonts P Reginster JY Bruyère O. Consequences of maternal postpartum depression: a systematic review of maternal and infant outcomes. Women’s Health (London). (2019) 15:1745506519844044. 10.1177/1745506519844044 PMC6492376 31035856

[56] Sockol LE Battle CL. Maternal attitude, depression, and anxiety in pregnant and postpartum multiparous women. Arch Women’s Ment Health. (2015) 18(4):585–93. 10.1007/s00737-015-0510-3 25712795

[57] Stapleton LR Schetter CD Westling E Rini C Glynn LM Hobel CJ Perceived partner support in pregnancy predicts lower maternal and infant distress. J Fam Psychol. (2012) 26(3):453–63. 10.1037/a0028332 22662772 PMC3992993

[58] Šebela A Hanka J Mohr P. Etiology, risk factors, and methods of postpartum depression prevention. Ceska Gynekologie. (2018) 83(6):468–73. 30848154

[59] Ukpong DI Owolabi AT. Postpartum emotional distress: a controlled study of Nigerian women after caesarean childbirth. J Obstet Gynaecol. (2006) 26(2):127–9. 10.1080/01443610500443386 16483968

[60] Upadhyay RP Chowdhury R Salehi A Sarkar K Singh SK Sinha B Postpartum depression in India: a systematic review and meta-analysis. Bull W H O. (2017) 95(10):706–17. 10.2471/BLT.17.192237 29147043 PMC5689195

[61] Weissman MM Pilowsky DJ Wickramaratne PJ Talati A Wisniewski SR Fava M Remissions in maternal depression and child psychopathology: a STAR*D-child report. JAMA. (2006) 295(12):1389–98. 10.1001/jama.295.12.1389 16551710

[62] Oates M. Suicide: the leading cause of maternal death. Br J Psychiatry. (2003) 183:279–81. 10.1192/bjp.183.4.279 14519602

[63] Sidebotham P Heron J, ALSPAC Study Team. Child maltreatment in the “children of the nineties”: a cohort study of risk factors. Child Abuse Negl. (2006) 30(5):497–522. 10.1016/j.chiabu.2005.11.005 16701895

[64] Lyons-Ruth K Lyubchik A Wolfe R Bronfman E. Parental depression and child attachment: hostile and helpless profiles of parent and child behavior among families at risk. In: Goodman SH Gotlib IH, editors. Children of Depressed Parents: Mechanisms of Risk and Implications for Treatment. Washington, DC: American Psychological Association (2002). p. 89–120. 10.1037/10449-004

[65] Beck CT. Predictors of postpartum depression: an update. Nurs Res. (2001) 50(5):275–85. 10.1097/00006199-200109000-00004 11570712

[66] Bloch M Schmidt PJ Danaceau M Murphy J Nieman L Rubinow DR. Effects of gonadal steroids in women with a history of postpartum depression. Am J Psychiatry. (2000) 157(6):924–30. 10.1176/appi.ajp.157.6.924 10831472

[67] Mercer RT. Becoming a mother versus maternal role attainment. J Nurs Scholarsh. (2004) 36(3):226–32. 10.1111/j.1547-5069.2004.04042.x 15495491

[68] Nelson AM. Transition to motherhood. J Obstet Gynecol Neonatal Nurs. (2003) 32(4):465–77. 10.1177/0884217503255199 12903696

[69] Rahman A Fisher J Bower P Luchters S Tran T Yasamy MT Interventions for common perinatal mental disorders in women in low- and middle-income countries: a systematic review and meta-analysis. Bull W H O. (2013) 91(8):593–601. 10.2471/BLT.12.109819 23940407 PMC3738304

[70] Stewart DE Robertson E Dennis CL Grace SL Wallington T. Postpartum Depression: Literature Review of Risk Factors and Interventions. Toronto: University Health Network Women’s Health Program (2003).

[71] Surkan PJ Kennedy CE Hurley KM Black MM. Maternal depression and early childhood growth in developing countries: systematic review and meta-analysis. Bull World Health Organ. (2011) 89(8):607–15. 10.2471/BLT.11.088187 PMC3150769 21836759

[72] Herba CM Glover V Ramchandani PG Rondon MB. Maternal depression and mental health in early childhood: an examination of underlying mechanisms in low- and middle-income countries. Lancet Psychiatry. (2016) 3(10):983–92. 10.1016/S2215-0366(16)30148-1 27650772

[73] Milgrom J Gemmill AW Bilszta JL Hayes B Barnett B Brooks J Antenatal risk factors for postnatal depression: a large prospective study. J Affect Disord. (2008) 108(1–2):147–57. 10.1016/j.jad.2007.10.014 18067974

[74] Parsons CE Young KS Rochat TJ Kringelbach ML Stein A. Postnatal depression and its effects on child development: a review of evidence from low- and middle-income countries. Br Med Bull. (2012) 101:57–79. 10.1093/bmb/ldr047 22130907

[75] Waqas A Rahman A Malik A Atif N Sikander S Roberts C Association of antenatal depression and postnatal depression with adverse maternal and infant outcomes in low- and middle-income countries: a systematic review and meta-analysis. J Affect Disord. (2020) 274:143–52. 10.1016/j.jad.2020.05.016

[76] Yim IS Tanner Stapleton LR Guardino CM Hahn-Holbrook J Dunkel Schetter C. Biological and psychosocial predictors of postpartum depression: systematic review and call for integration. Annu Rev Clin Psychol. (2015) 11:99–137. 10.1146/annurev-clinpsy-101414-020426 25822344 PMC5659274

[77] O’Hara MW McCabe JE. Postpartum depression: current status and future directions. Annu Rev Clin Psychol. (2013) 9:379–407. 10.1146/annurev-clinpsy-050212-185612 23394227

[78] Maharjan PL Lamichhane B. Prevalence and factors associated with postpartum depression among mothers attending maternal and child health clinics in Nepal. J Inst Med. (2012) 34(2):44–8.

[79] Subba R Budhathoki SS Shah R Shrestha N Adhikari SR. Prevalence of postpartum depression and associated factors among mothers in Nepal. BMC Pregnancy Childb. (2018) 18:xxx.

[80] Weobong B Ten Asbroek AH Soremekun S Manu AA Owusu-Agyei S Prince M Association between probable postpartum depression and increased infant mortality and morbidity: findings from the DON population-based cohort study in rural Ghana. BMJ Open. (2014) 4:e004838. 10.1136/bmjopen-2014-004838 26316646 PMC4554911

[81] Upadhyay RP Chowdhury R Salehi A Sarkar K Singh SK Sinha B Postpartum depression in India: a systematic review and meta-analysis. Bull World Health Organ. (2011) 89(7):522–30. 10.2471/BLT.10.084103 PMC5689195 29147043

[82] Yadav DK Shams R Khan AF Azam H Anwar M Anwar T Depression among postpartum women in Nepal: a hospital-based study. Cureus. (2020) 12(12):e12216. 10.7759/cureus.12216 33489623 PMC7815271

[83] Kline RB. Principles and Practice of Structural Equation Modeling. 4th ed New York, NY: The Guilford Press (2015).

[84] Fabrigar LR Wegener DT MacCallum RC Strahan EJ. Evaluating the use of exploratory factor analysis in psychological research. Psychol Methods. (1999) 4(3):272–99. 10.1037/1082-989X.4.3.272

