# Supplementary material for: Emotional distress among postpartum women in central Nepal: a cross-sectional study using structural equation modeling
Source: Front Glob Womens Health. 2026 Mar 27;7:1723556. doi: 10.3389/fgwh.2026.1723556 (PMC13066223; doi:10.3389/fgwh.2026.1723556)
Supplement: Supplementary file 1 [file Table1.docx]

**Supplementary Table S1.**

| *factors* |  |  | *participants* |  |  |  |
| --- | --- | --- | --- | --- | --- | --- |
|  | n=381 | *G1: Non-ED*  n=228(59.8%) | *G2:*  *EPDS ≥ 10*  n=103(27%) | *ED*  n=153(40.2%)  *G3:*  *GHQ-12 ≥ 2*  n=17(4.5%) | *G4: EPDS ≥10 &*  *GHQ-12 ≥ 2*  n=33(8.7%) | *p* |
| *＜Four-point scale items＞ (Mean±SD)*  *Is your child currently healthy?*  *Are you satisfied with your child's gender?*  *Before you gave birth, did you have experience caring for a baby?*  *Do you have knowledge of childcare?*  *Have you been getting sufficient sleep recently?*  *Do you receive help from your husband in raising children?*  *Do you receive help from other family members (besides your husband) in raising children?*  *Do you have a good relationship with your husband?*  *Are you satisfied with the married life you have with your husband?*  *Do you have a good relationship with your husband's mother?*  *Does your husband drink excessive amounts of alcohol?*  *Has your husband ever physically attacked you or thrown something at you that might hurt you?*  *Has your husband ever forced you to engage in sexual activity against your will?*  *Has your husband ever insulted or shouted at you in a way that made you feel bad about yourself?*  *Does your husband work away from home and leave the house for long periods of time?*  *Have you experienced stressful life events in recent years (e.g., death of a family member)?* | 3.83 (0.41)  3.99(0.08)  2.83(1.34)  3.15(1.07)  3.61(0.70)  3.30(1.02)  3.11(1.33)  3.98(0.19)  3.95(0.27)  3.54(0.95)  1.18(0.47)  1.01(0.13)  1.00(0.05)  1.10(0.30)  1.43(0.74)  1.22(0.76) | 3.86(0.37)  4.00(0.06)  2.82(1.34)  3.17(1.06)  3.68(0.64)  3.34(1.02)  3.23(1.28)  4.00(0.00)  3.96(0.29)  3.61(0.90)  1.18(0.48)  1.01(0.09)  1.00(0.06)  1.06(0.24)  1.45(0.77)  1.19(0.73) | 3.81(0.46)  3.99(0.09)  2.79(1.35)  3.05(1.12)  3.67(0.63)  3.41(0.96)  3.05(1.35)  3.99(0.09)  3.97(0.17)  3.57(0.94)  1.08(0.30)  1.03(0.21)  1.00(0.00)  1.12(0.32)  1.42(0.74)  1.16(0.65) | 3.82(0.39)  4.00(0.00)  2.71(1.26)  3.12(0.99)  3.24(0.97)  3.12(0.85)  3.00(1.41)  3.82(0.72)  3.94(0.24)  3.69(0.70)  1.35(0.78)  1.00(0.00)  1.00(0.00)  1.12(0.33)  1.24(0.56)  1.71(1.31) | 3.70(0.46)  3.97(0.17)  3.15(1.32)  3.39(0.96)  3.21(0.96)  2.79(1.11)  2.55(1.41)  3.85(0.36)  3.82(0.39)  3.00(1.19)  1.33(0.54)  1.00(0.00)  1.00(0.00)  1.30(0.46)    1.45(0.66)  1.33(0.92) | 0.11^C^  0.44^C^  0.47^C^  0.42^C^  <0.01^d^ *G1,G2－G4*  <0.01^d^ *G1,G2－G4*  <0.05^d^ *G1—G4*  <0.001^d^ *G1—G3, G4*  <0.001^d^ *G1,G2－G4*  <0.01^d^ *G1,G2－G4*  <0.01^d^ *G2－G4*  0.70^C^  0.87^C^  <0.001^d^ *G1,G2－G4*  0.64^C^  <0.05^d^ *G1,G2－G3* |

Comparison of childcare situations, relationships and support, stress due to husbands’ behavior, and stressful life events by emotional distress classification (n=381)

c：Kruskal-Wallis test (Bonferroni was performed when p < 0.05) d：Multiple comparison by Bonferroni (only significant group differences shown)

Groups: G1 =Non-ED; G2 = EPDS ≥ 10 only; G3 = GHQ-12 ≥ 2 only; G4 = EPDS ≥ 10 & GHQ-12 ≥ 2.

**Supplementary Table S2.** Correlation coefficients among latent variables

| latent variables | Ⅰ | Ⅱ | Ⅲ | Ⅳ | Ⅴ | Ⅵ | Ⅶ |
| --- | --- | --- | --- | --- | --- | --- | --- |
| Ⅰ：Child-caring Experience  Ⅱ：Mother’s Economic Strength  Ⅲ：Age of Parents  Ⅳ：Extended Family Supporters  Ⅴ：Emotional Distress  Ⅵ：Receive Help from Husband  Ⅶ：Satisfaction with Married Life | 1.00  -.01  .38^**^  -.26^***^  .04  -.05  -.16^**^ | 1.00  .19^***^  .17^**^  -.16^**^  .01  -.04 | 1.00  -.14^*^  .09  .03  -.19^*^ | 1.00  -.14^**^  .12^*^  .13^*^ | 1.00  -.15^**^  -.67^**^ | 1.00  .05 | 1.00 |

Pearson’s correlation coefficients are shown for the relationships among latent variables.

*p < 0.05, **p < 0.01, ***p < 0.001.
